# Do Adverse Childhood Experiences (ACEs) Influence getting recommended vaccines for Florida Residents ages 50+?

**DOI:** 10.1093/geroni/igaf122.3916

**Published:** 2025-12-31

**Authors:** Stephanie Clervil, Roseline Ukenenye, Sandro Louis, Priscilla Ryder, Aymara Diaz

**Affiliations:** Miami Dade College, Miami, Florida, United States; Howard University, Washington, District of Columbia, United States; Miami Dade College, Miami, Florida, United States; Larkin University College of Pharmacy, Miami, Florida, United States; Larkin University, Miami, Florida, United States

## Abstract

Adverse childhood experiences (ACEs) are traumatic events that occur before the age of 18, including abuse, neglect, and family dysfunction. ACEs are associated with many adult physical and mental health outcomes (e.g., cardiovascular disease, diabetes, cancer, arthritis, depression and anxiety, premature death). ACEs are also associated with negative health behaviors (e.g., tobacco use, binge drinking). Little is known about the effects of ACEs on other health behaviors, such as getting recommended immunizations. This investigation used data from the 2023 Behavioral Risk Factor Surveillance System (BRFSS) from Florida for respondents ages 50 and older. ACEs were summed to give a score of 0 (no adverse experiences) to 8 (maximum number). The typical respondent was a White, retired, female, high school graduate who received Medicare. 3679 (44.6%) and 4597 (55.0%) reported getting shingles and pneumonia vaccines, respectively. The median number of ACEs was 1, with 0 being the most common value. In bivariate analysis, ACEs were highly associated with being vaccinated (Chi-square (8) = 26.557, p < 0.001)—the higher the number of ACEs, the smaller the percentage vaccinated. ACEs lost significance in binary logistic models for receiving the vaccines. Factors that increased the odds of being vaccinated were having a personal health care provider and being retired, while being Black or Hispanic (both vaccines), being Asian (pneumonia vaccine only), and reporting poor mental health decreased the odds of vaccination, adjusting for education and home ownership. The relationship between ACEs and immunization may be mediated by socioeconomic factors.

